# 

**Published:** 1895-01

**Authors:** 


### CONTENTS.

ORIGINAL ARTICLES:

Veterinary Education in Relation to the Practice of Veterinary Medicine in New York State. By PROF. A. LIAUTARD, M.D., V.M. . . . . . . . . . 1

Purpura Hemorrhagica: Also Called Eruptic Fever, Petechial Fever, Anasarca, etc. By J. W. SALLADE, V.S. . . . . . . . . . . . . 9

Veterinary Science. By L. M. KLUTTS, V.S. . . . . . . . . . . 14

Tuberculosis in Relation to Animal Industry and Public Health. Its Prevalence and Relative Importance. By ANDERSON CROWFORTH, V.S. . . . . . . 20

SELECTIONS:

A State Veterinarian for California . . . . . . . . . . . 36

Review of Sanitary Measures . . . . . . . . . . . . . 39

REVIEWS:

Pathology and Therapeutics of the Domestic Animals . . . . . . . 41

Every Man His Own Horse Doctor . . . . . . . . . . . . 41

EDITORIAL:

Prohibition of Importation of American Cattle—Obituary—Veterinary Legislation in California—United States Veterinary Medical Association—Correction . . 42

SOCIETY PROCEEDINGS:

Massachusetts Veterinary Association . . . . . . . . . . . 45

Ontario Veterinary College Society . . . . . . . . . . . 47

Ontario Veterinary Association . . . . . . . . . . . . 47

Keystone Veterinary Medical Association . . . . . . . . . . 49

Veterinary Medical Association of New Jersey . . . . . . . . . 50

Missouri Valley Veterinary Association . . . . . . . . . . 51

Schuylkill Valley Veterinary Medical Association . . . . . . . . 52

American Institute Farmers' Club . . . . . . . . . . . 52

United States Veterinary Medical Association . . . . . . . . . 53

California State Veterinary Medical Association . . . . . . . . . 45

NEWS ITEMS—EQUINE FOODS—ITEMS OF INTEREST—COLLEGE NOTES—CORRESPONDENCE . . . . . . . . . . . . . . . . . 57–64

